# Case Report: A Novel Mutation in the Mitochondrial *MT-ND5* Gene Is Associated With Leber Hereditary Optic Neuropathy (LHON)

**DOI:** 10.3389/fneur.2021.652590

**Published:** 2021-03-25

**Authors:** Martin Engvall, Aki Kawasaki, Valerio Carelli, Rolf Wibom, Helene Bruhn, Nicole Lesko, Florian A. Schober, Anna Wredenberg, Anna Wedell, Frank Träisk

**Affiliations:** ^1^Department of Molecular Medicine and Surgery, Karolinska Institutet, Stockholm, Sweden; ^2^Centre for Inherited Metabolic Diseases, Karolinska University Hospital, Stockholm, Sweden; ^3^Hopital Ophtalmique Jules Gonin, Fondation Asile des Aveugles, University of Lausanne, Lausanne, Switzerland; ^4^Programma di Neurogenetica, IRCCS Istituto delle Scienze Neurologiche di Bologna, Bologna, Italy; ^5^Department of Biomedical and Neuromotor Sciences (DIBINEM), University of Bologna, Bologna, Italy; ^6^Department of Medical Biochemistry and Biophysics, Karolinska Institutet, Stockholm, Sweden; ^7^Department of Clinical Neuroscience, Division of Eye and Vision, St. Erik Eye Hospital, Karolinska Institutet, Solna, Sweden; ^8^Department of Neuro-Ophthalmology, St.Erik Eye Hospital, Solna, Sweden

**Keywords:** leber hereditary optic neuropathy, optic neuropathy, MT-ND5, mitochondrial DNA, case report, complex 1, LHON

## Abstract

Leber hereditary optic neuropathy (LHON) is a mitochondrial disease causing severe bilateral visual loss, typically in young adults. The disorder is commonly caused by one of three primary point mutations in mitochondrial DNA, but a number of other rare mutations causing or associated with the clinical syndrome of LHON have been reported. The mutations in LHON are almost exclusively located in genes encoding subunits of complex I in the mitochondrial respiratory chain. Here we report two patients, a mother and her son, with the typical LHON phenotype. Genetic investigations for the three common mutations were negative, instead we found a new and previously unreported mutation in mitochondrial DNA. This homoplasmic mutation, m.13345G>A, is located in the *MT-ND5* gene, encoding a core subunit in complex I in the mitochondrial respiratory chain. Investigation of the patients mitochondrial respiratory chain in muscle found a mild defect in the combined activity of complex I+III. In the literature six other mutations in the *MT-ND5* gene have been associated with LHON and by this report a new putative mutation in the *MT-ND5* can be added.

## Introduction

Leber hereditary optic neuropathy (LHON) usually manifests as a sequential subacute optic neuropathy. A pathogenic mutation in the mitochondrial DNA (mtDNA), in conjunction with environmental and possibly other genetic factors, leads to the disruption and progressive loss of small caliber retinal ganglion cells in the papillo-macular bundle (1–3). Typically the patient with LHON presents with monocular loss of visual acuity in parts of the central visual field. Involvement of the second eye occurs within 1 year, in most cases already within 6–8 weeks. The optic disc appearance may at first assessment be normal or show blurred margins and telangiectatic microangiopathy. Over the following weeks or months vision continues to deteriorate as a result of the progressive loss of retinal ganglion cells and atrophy of the optic nerves (3). LHON is a rare cause of optic neuropathy, with a prevalence of about 1 in 30,000 to 1 in 50,000 people in the population (4–6). Male gender is clearly associated with an increased risk of the disease, as roughly 50% of males and only 10% of female LHON carriers develop visual loss (7). Other likely risk-factors are exposure to tobacco and alcohol (8) as well as modifying genetic factors that may regulate disease penetrance (2, 9). If there is no family history of LHON, there is an inherent risk that the clinician will falsely suspect the patient with LHON to have optic neuritis since both disorders often manifest at age 15–35 years. However, the painless continuous progression of visual loss without recovery does not follow the evolution of a typical optic neuritis, and therefore such a presentation requires an early and thorough work-up to exclude other causes of acute optic neuropathy.

In LHON three common point mutations in mtDNA constitute the primary pathogenic mutations, m.11778G>A (*MT-ND4*), m.3460G>A (*MT-ND1*) and m.14484T>C (*MT-ND6*). Together they account for 90% of symptomatic patients with LHON. The proteins encoded by these genes are crucial subunits for complex I of the mitochondrial respiratory chain (7, 10).

Progress in DNA-sequencing techniques of both nuclear and mitochondrial genes, in conjunction with increasing awareness of mitochondrial disease, has led to many new mutations reported in association with clinically recognized phenotypes of mitochondrial diseases, including LHON. The collective prevalence of adult mitochondrial disease now makes them among the most common adult form of inherited neurological disorders, when including disease-causing mutations in both the mitochondrial and nuclear genome (11). More information is continuously being reported about previously undescribed mtDNA mutations and their phenotypic features.

Mitomap, a database of human mitochondrial DNA variations, reports 19 primary mtDNA mutations which are now associated with the LHON phenotype and 18 others which are candidate mutations for singleton or single families (12).

Herein, we report a mother and her adult son who presented in the same year with a severe bilateral optic neuropathy. There was no known family history of optic neuropathy. Testing for the three primary LHON mutations was negative, but whole genome sequencing (WGS) analysis of DNA from muscle targeting both nuclear genes causing mitochondrial and other metabolic diseases and mtDNA identified the same unique homoplasmic mutation in both subjects, m.13345G>A, p.(Ala337Thr) in *MT-ND5*. No other disease-causing mutation was found that could explain the phenotype.

## Case Report

### Case 1 (Proband)

**Figure 1:**
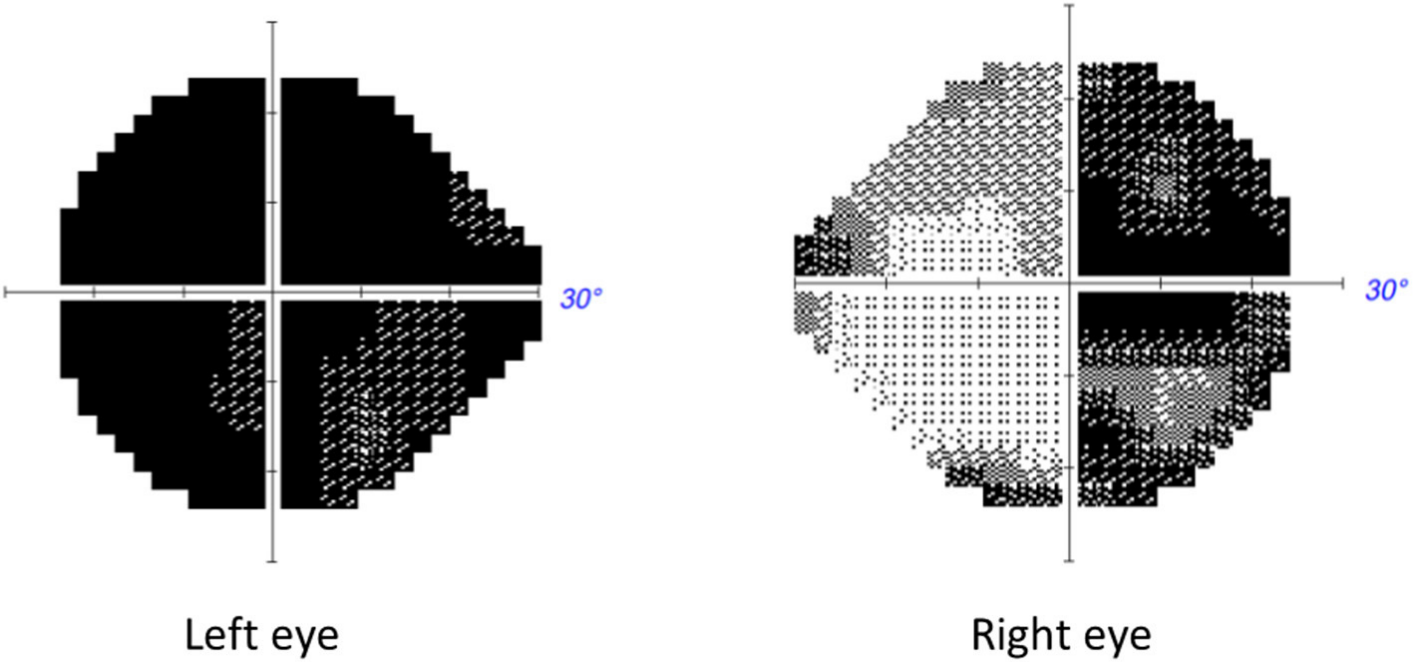
Automated Humphrey visual field of patient described in Case 1 performed 3 weeks from symptomatic decline of vision in the second eye, i.e., the right eye. A temporal defect is noted in the RE and a central defect in the LE.

A 49 year old woman came to the eye clinic for rapid and painless visual loss in her right eye (RE) over 3 weeks. Prior to right eye visual loss, she had noted deteriorating vision in her left eye (LE) but could not be more specific about the time of onset. Review of prior records showed that 1 month prior to presentation, her optician had documented best corrected visual acuity (BCVA) as 0.8 in the RE and 0.05 in the LE. She was a consumer of tobacco mainly in the form of snuff (oral smokeless tobacco) and she occasionally smoked 5–10 cigarettes a day. She denied regular use of alcohol but according to medical records she had a history of periodic high consumption of alcohol. She had also been prescribed vitamin B12 daily. Past medical history was otherwise unremarkable. The patient has one son and two sisters of which one has a daughter. The patient's mother had died from malignant melanoma and the maternal grandmother from ruptured aortic aneurysm. None of the relatives known to the patient had any history of visual problems, dementia, muscle disease, diabetes or hearing problems apart from one sister having some hearing loss after a rubella infection in childhood. On examination, BCVA was 0.13 in the RE and hand motion in the LE Humphrey® automated visual fields 24-2 (Carl Zeiss Meditec.Inc®, Jena) showed a temporal defect encroaching fixation in the RE and a large central scotoma in the LE (Figure 1). A relative afferent pupillary defect was observed in the LE. Funduscopy showed slight temporal pallor of the optic disk and there was an epiretinal membrane in the LE.

The rapidly progressing deep central scotoma in one eye combined with a temporal hemidefect in the other eye suggested pathology at the chiasm. The MRI showed increased signal on T2 and FLAIR sequences without contrast enhancement in the central optic chiasm, in the optic tracts and in the intracranial portion of both optic nerves. There was no signal abnormality in the brain or brainstem. The patient underwent extensive investigations for various causes of an acute optic neuropathy and corticosteroid therapy was initiated for presumed neuromyelitis optica. A lumbar puncture was performed for analysis of cerebrospinal fluid including electrophoresis. Other work-up for inflammatory neuropathy included CT scan of the thorax and serum analysis for angiotensin converting enzyme and many autoantibodies: S-ANA, antibodies against the S-nucleosome, S-Ribosomal P, S-RNP68, S-Scl-70, S-Sm, S-SmRNP, SS-A, SS-B, Centromer, Jo-1, dsDNA, antiMOG and Aquaporin 4 in serum, of which all were negative. Since the patient's vision did not improve after 5 days of corticosteroids, the severity of visual loss prompted further treatment with plasmapheresis. This also failed to improve vision.

The history of tobacco/alcohol use and vitamin B12 vitamin substitution raised consideration for malnutritional optic neuropathy, but levels of folic acid, vitamin B6 and cobalamin were found to be within normal limits. Intravenous multivitamin injections were however given *ex juvantibus*. Three weeks after presentation, OCT (Carl Zeiss Meditec.Inc®, Jena) showed significant thinning of the ganglion cell-internal plexiform layer thickness predominantly in the nasal parts with the average thickness of 65 μm in the RE and 82 μm in the LE. The peripapillary retinal nerve fiber layer (pRNFL) thickness was still preserved (96 μm in RE and 122 μm in LE) but artefactually augmented in the LE due to missegmentation due to the epiretinal membrane. A fullfield ERG was normal, confirming intact retinal photoreceptor function.

Even though the family history was negative, the sequential visual loss and absence of response to treatment for optic neuritis suggested mitochondrial origin, but since the three most common mutations associated with LHON were negative we pursued more extensive molecular analysis, see below.

### Case 2: Son

The 19 year old son of the proband was referred to the eye clinic in a neighboring town. He had not been in contact with his mother for several years, but had heard through a relative that she was under investigation for loss of vision. In the preceding 2 months he had experienced progressive painless visual deterioration in both eyes and the news of similar symptoms in his mother prompted him to seek medical advice. The patient was smoking ~10 cigarettes a day and had a history of recreational drug use. During the few months prior to visual loss, he had consumed more alcohol than usual and had used cannabis more than once a week. He denied ingestion of other toxic substances in the months preceding visual loss.

On examination BCVA was 0.04 in the RE and 0.02 in the LE with bilateral central scotomas and temporal pallor of both optic nerves. There was no significant temporal thinning in the pRNFL on OCT but at this stage the average pRNFL was still relatviely preserved at 92 μm in the RE and 94 μm in the LE.

When the treating ophthalmologist discovered that the patient's mother was under investigation for LHON, there was an immediate suspicion of the same diagnosis. MRI of the brain and orbit was normal. Routine laboratory tests including serum vitamin B screening, ANA and antibodies against aquaporin-4 and myelin oligodendrocyte glycoprotein were normal. The three most common mutations for LHON were negative.

**Figure 2:**
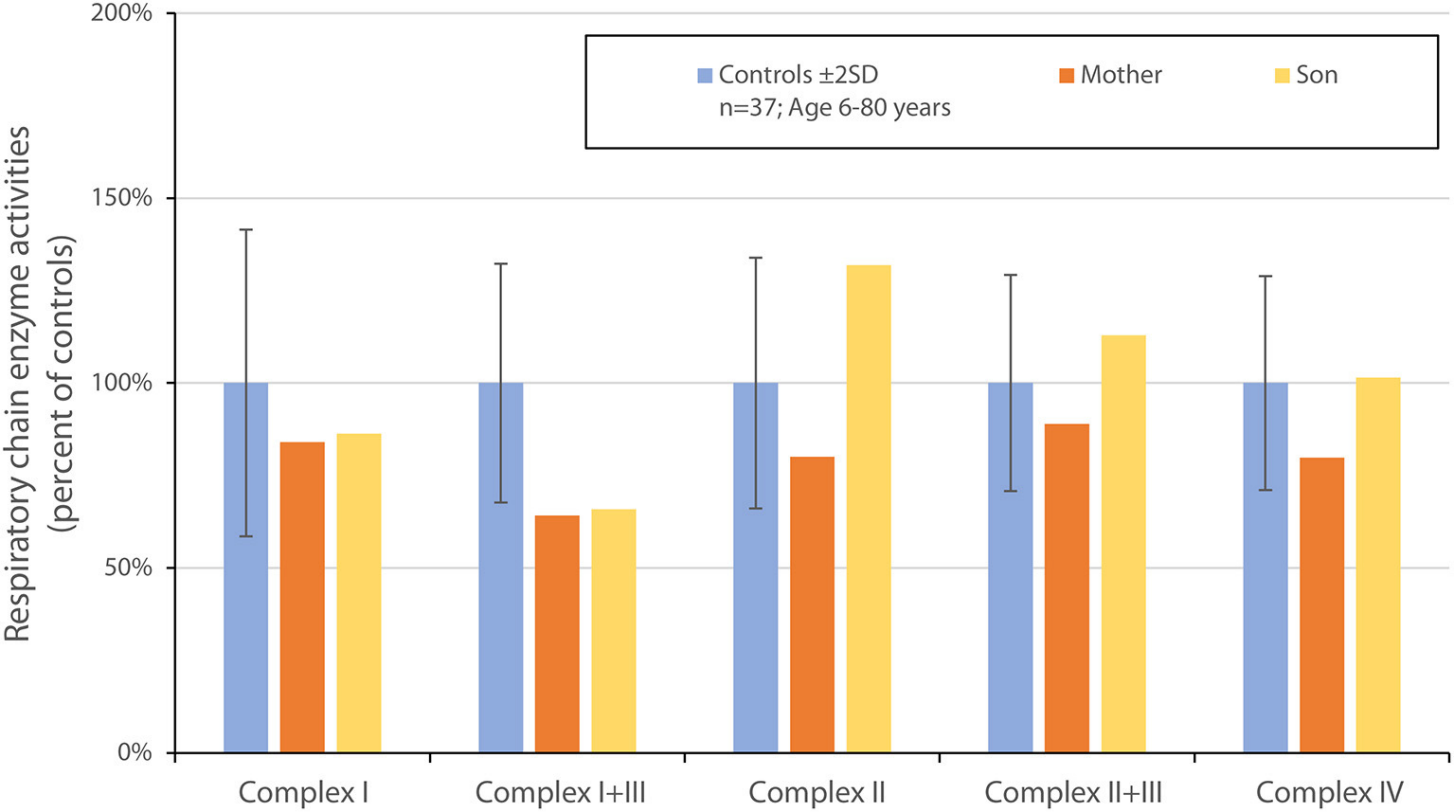
Graphic of respiratory chain enzyme activities in the muscle biopsy of the two patients described in Case 1 and Case 2, as compared to normal controls. There is a mild reduction in complex I + III.

In both cases we suspected mitochondrial optic neuropathy and extended investigations by performing muscle biopsies for mitochondrial biochemical investigations and targeted whole genome sequencing (WGS) to look for mitochondrial as well as all other inherited optic neuropathies. A percutaneous muscle specimen was obtained from the anterior tibial muscle using a conchotome. Mitochondria were isolated from muscle and mitochondrial ATP production rate and respiratory chain enzyme activities were determined as previously described (13). The muscle biopsy showed normal mitochondrial ATP production rates (data not shown). Measurements of the respiratory chain enzyme activities revealed a mild reduction in complex I+III in both the patient and her son while all other activities (including complex I) were found to be within the reference range (Figure 2).

**Figure 3:**
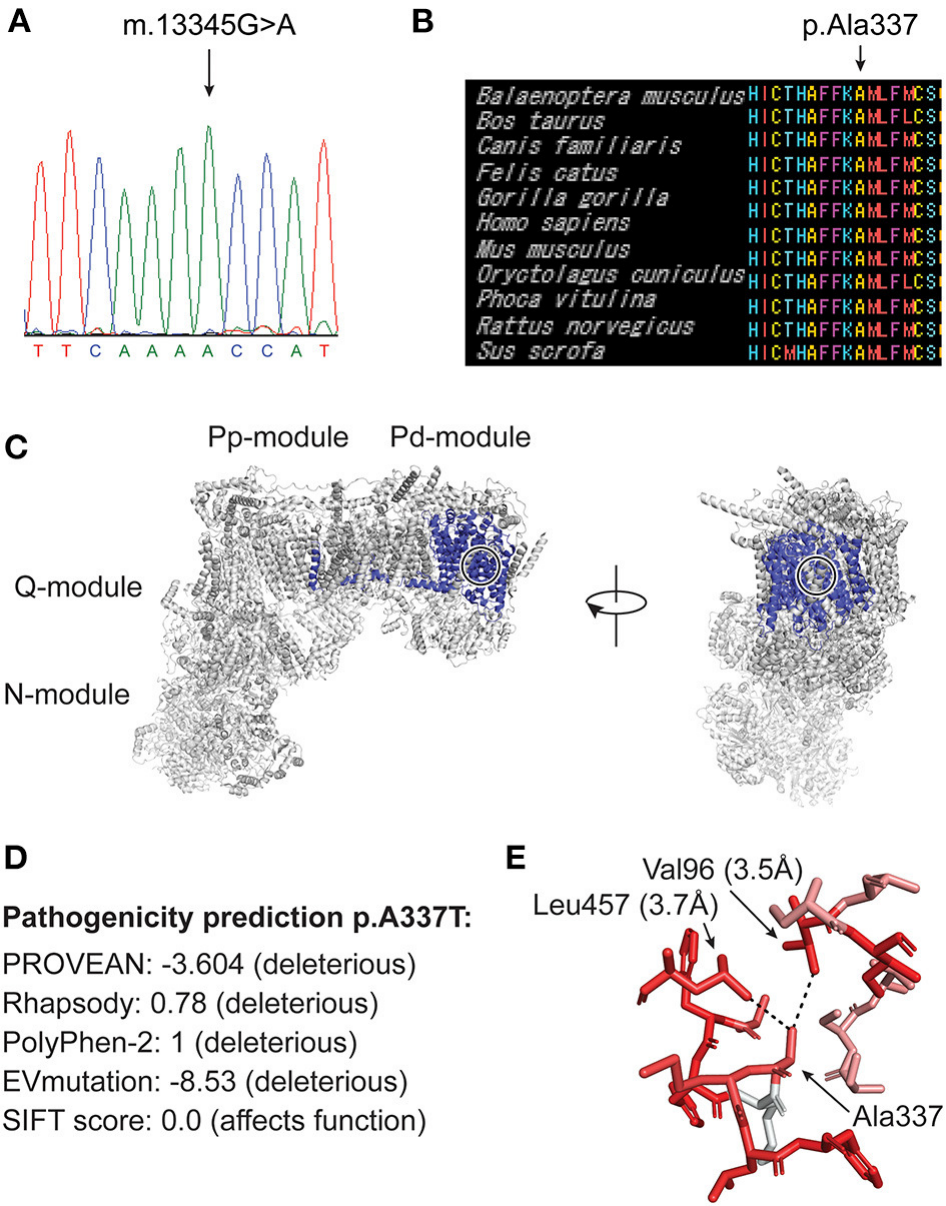
**(A)** Sanger sequencing of DNA from skeletal muscle from case 1 showing homoplasmic m.13345G>A mutation. **(B)** MtSNP Database mtSAP alignment of 11 mammals showing that the residue Ala337 and the adjacent aminoacids are very well-conserved (all 61 mammals in the database have alanine at this position). **(C)** A human cryo-EM structure of complex I (PDB:5XTD), with ND5 highlighted in blue. Frontal (right) and lateral (left) view. Ala337 is buried inside the protein structure and its position is indicated with black/white circles. **(D)** Pathogenicity prediction score of different tools. PROVEAN, Rhapsody, PolyPhen-2, EVmutation and SIFT. **(E)** A337 is shown in the above structure with PyMOL version 2.3.4. (Schrödinger, LLC.) with its closest interactors Leu457 and Val96. Dotted lines mark the quantified distances between the side chain atoms. The color codes for hydrophobicity, whereby red is strongly hydrophobic and white is strongly hydrophilic. This indicates that Ala337 is in a strongly hydrophobic environment. The plugin color_h was used on the basis of a previously established hydrophobicity grading (15).

Blue Native Gel Electrophoresis showed normal amount and composition of complexes I-V (data not shown). WGS of DNA extracted from skeletal muscle was performed to a sequencing depth of 30x mean coverage using a NovaSeq 6000 sequencing instrument (Illumina) after library preparation with NxSeq AmpFREE Low DNA Library Kit (Lucigen). This was followed by in-house bioinformatics analysis, using the mutation identification pipeline (MIP) as earlier described (14). Analysis of all known nuclear disease genes associated with optic neuropathies (for gene list see Supplementary File 1), mitochondrial and metabolic diseases and mtDNA detected the mutation m.13345G>A, p.(Ala 337Thr) in the mitochondrially encoded NADH:ubiquinone oxidoreductase core subunit 5 (*MT-ND5*) in homoplasmy and was assumed to be related to the optic neuropathy (Figure 3A). No other disease-causing mutations that could explain the phenotype were identified. Sanger sequencing of DNA extracted from urinary epithelial cells and blood also showed the mutation in homoplasmy.

After the detection of a homoplasmic mtDNA mutation, additional investigations for other clinical manifestations of mitochondrial disease including echocardiography, electrocardiography, audiography and electromyography were performed. All were normal apart from a slight sublinical decline in the high frequencies in the audiogram of both ears in the mother.

**Figure 4:**
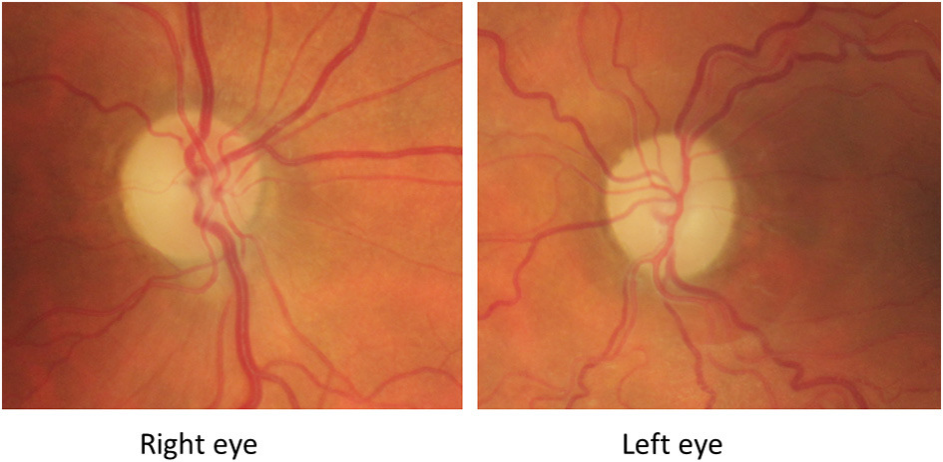
Fundus-photography of patient described in Case 1 taken 5 months from presentation to the ophthalmic clinic. There is bilateral pallor of the optic disk and a non-related epiretinal membrane in the left eye 5 months after presentation.

At this stage both mother and son were diagnosed with LHON. They were adviced to refrain from smoking and use of alcohol and also prescribed Idebenone 900 mg daily. Regrettably this did not improve vision and after a year the treatment was stopped. During the months following onset, optic atrophy developed (Figure 4). Sixteen months from presentation, the BCVA of the mother was 0.04 in the RE and 0.02 in the LE and deep central scotomas were present on Humphrey perimetry 20 months after the first visit, BCVA of the son was in the RE 0.04 and in the LE 0.04 with deep central scotomas still present. OCT (Topcon®, Dublin, CA) showed severe thinning of the pRNFL; 50 microm in the RE and 48 microm in the LE consistent with bilateral optic atrophy.

## Discussion

Although the age at symptomatic onset is broad, patients with LHON typically lose vision between the ages of 15 and 35 years. In this report of a familial optic neuropathy, the mother at age 49 years and her son at age 19 years experienced onset of visual loss within the same 6 month period. Because of this curious coincidence, a shared external factor which might have precipitated symptomatic expression of the mtDNA mutation was suspected. Specifically, we wondered about exposure to similar toxic substances or illicit drugs. We investigated the background of tobacco, alcohol and other drug consumption in both patients. There was no evidence of home-distilled alcohol consumption from a mutual supplier nor corroboration of purchase of similar recreational drugs from the same source. In fact, the son was living in Spain for several months just before and at the time of his visual decline, while his mother had stayed in Sweden. Thus, the only identified shared external factor was tobacco use (snuff and cigarettes in the mother and cigarettes in the son) and commercially-purchased alcohol consumption. The striking similarity and timing of the visual loss in our patients suggest that the tobacco and alcohol use could influence penetrance of the same underlying mtDNA mutation.

The homoplasmic mutation m.13345G>A, p.(Ala337Thr) in the *MT-ND5* gene found in our two patients is not previously described in patients with LHON or other mitochondrial disorders. The mutation is reported in heteroplasmy in one individual in the Helix mitochondrial database of 195,983 sequences (16), but is not present in 51673 GenBank sequences according to Mitomap (12) and not in 2,704 sequences in the Human Mitochondrial Genome Database (mtDB) (17). The mtSNP Database mtSAP (18) shows that the amino acid residue affected by the mutation is evolutionarily very well conserved. All 61 mammals in the database have alanine in that position (Figure 3B). The mutation is located in the membrane arm of complex I (Figure 3C) and is predicted to be pathogenic by the prediction tools PROVEAN (19), Rhapsody (20), Polyphen-2 (21), EVmutation (22) and SIFT (23) (Figure 3D). Ala337 is buried inside the ND5 protein structure (Figure 3C) in a human cryogenic electron microscopy (cryo-EM) structure of complex I (24). Alanine is a small apolar amino acid, and Ala337 is in close proximity to at least two highly hydrophobic residues Val96 and Leu457 in ND5 (Figure 3E). We suggest that any other amino acid at this position will be too large and will affect the apolar environment, thus changing the conformation of surrounding side chains.

Mutations in *MT-ND5* have been associated with the LHON phenoype in several other families or single patients (25–28) and also in other phenotypic syndromes including Leigh syndrome, mitochondrial encephalopathy with lactic acidosis and stroke-like episodes (MELAS) and myoclonic epilepsy with ragged red Fibers (MERRF) (29, 30). In other cases of *MT-ND5* mutations in LHON defective assembly could be demonstrated (31). Given the high energy demand of retinal cells, including ganglion cells, visual loss is a common manifestation of various mitochondrial disorders (18). Among patients having optic neuropathy, most are associated with mutations affecting complex I subunits. *MT-ND5*, the subunit affected in our two patients, is one of the core subunits of complex I and is closely associated with one of the proton pumping pores (10). In our subjects we could document a modest but significant reduction of the combined activity of complex I + III, but not when complex I was measured in isolation. Blue native gel electrophoresis showed no signs of deficiency or disruption of neither complex I or of complex II-V. Our two patients did not have any other symptoms apart from visual loss and all laboratory investigations showed no indication of dysfunction of other organ systems.

In summary, we report a novel mtDNA mutation associated with subacute onset of bilateral optic neuropathy in a mother and her adult son. This mutation in combination with the typical clinical findings suggests it is a new putative LHON-mutation.

## Data Availability Statement

The datasets presented in this article are not readily available because this would jeopardize patient integrity. Requests to access the datasets should be directed to the corresponding author.

## Ethics Statement

Ethical review and approval was not required for the study on human participants in accordance with the local legislation and institutional requirements. The patients/participants provided their written informed consent to participate in this study. Written informed consent was obtained from the individual(s) for the publication of any potentially identifiable images or data included in this article.

## Author Contributions

AK, FT, ME, and VC contributed to conception of the manuscript. AK, FT, and ME collected the original data. HB, NL, and ME performed the genetic and bioinformatic analysis. RW, HB, and NL performed the mitochondrial biochemical investigations. ME performed the muscle biopsies. AK, ME, FT, RW, HB, and VC drafted the manuscript with table and figures. AWe and AWr developed the sequencing and bioinformatic platforms and revised the manuscript critically. All authors contributed to the article and approved the submitted version.

## Conflict of Interest

The authors declare that the research was conducted in the absence of any commercial or financial relationships that could be construed as a potential conflict of interest.
