# Supplementary material for: Case Report: A Novel Mutation in the Mitochondrial MT-ND5 Gene Is Associated With Leber Hereditary Optic Neuropathy (LHON)
Source: Front Neurol. 2021 Mar 25;12:652590. doi: 10.3389/fneur.2021.652590 (PMC8027302; doi:10.3389/fneur.2021.652590)
Supplement: Supplementary file 1 [file Data_Sheet_1.pdf]

Supplemental file 1

Nuclear genes associated with optic nerve disease analyzed for variants

AAAS, AARS1, ABCA4, ABCC6, ABCG5, ABCG8, ABHD12, ACADS, ACER3, ACO2, ACOX1, ACTB, ACTG1, ACTL6A, ACTL6B, ADA2, ADAM22, ADAR, AFF4, AFG3L2, AGBL5, AGXT, AHI1, AHR, AIFM1, AIPL1, AKT1, ALDH1A3, ALG13, ALG3, ALMS1, AMPD2, ANKRD11, ANTXR1, AP1S2, AP3B2, APC, APOB, ARHGEF18, ARHGEF2, ARL13B, ARL2BP, ARL3, ARL6, ARNT2, ARSA, ARV1, ARVCF, ARX, ASB10, ASPA, ASXL1, ATAD3A, ATIC, ATN1, ATP1A3, ATP6V1A, ATRX, ATXN1, ATXN7, AUH, B3GALNT2, B3GALT6, B3GLCT, B4GAT1, B9D1, B9D2, BAP1, BBS2, BCAP31, BCOR, BCORL1, BEST1, BLOC1S3, BOLA3, BPTF, BRAF, BRAT1, BTD, C19orf12, C2CD3, C4A, C8orf37, CA2, CA4, CACNA1A, CACNA1B, CACNA1F, CASK, CASR, CBS, CC2D2A, CCDC22, CCDC88A, CCR1, CDC42, CDHR1, CDK19, CDON, CENPF, CEP290, CEP55, CERKL, CFAP410, CHD7, CHN1, CHSY1, CISD2, CKAP2L, CLCC1, CLCN2, CLCN7, CLN3, CLP1, CLRN1, CLTC, CNGA1, CNGB1, CNKSR2, CNNM4, CNOT3, COA8, COG6, COL18A1, COL4A1, COMT, COX15, COX7B, CPAMD8, CRB1, CRPPA, CRX, CSF1R, CSPP1, CTC1, CTNNB1, CYFIP2, CYP1B1, CYP27A1, CYP7B1, DAG1, DALRD3, DARS2, DDB2, DDHD2, DDX11, DDX6, DENND5A, DGUOK, DHCR7, DHDDS, DHX38, DIAPH1, DKC1, DNAJC19, DNAJC30, DNM1, DNM1L, DNMT1, DNMT3A, DOCK3, DOCK6, DPM1, DPM2, DPYD, DYRK1A, EBP, ECHS1, EEF1A2, EIF2B1, EIF2B2, EIF2B3, EIF2B4, EIF2B5, ELOVL1, ELP1, EMC1, EPG5, EPRS1, ERAP1, ERCC1, ERCC2, ERCC3, ERCC4, ERCC5, ERCC6, ERCC8, ERF, ESPN, EXOC8, EYS, FA2H, FAM111A, FAM161A, FANCB, FANCI, FAS, FBXW11, FCSK, FDFT1, FDX2, FDXR, FGF12, FGFR1, FGFR2, FGFR3, FH, FKRP, FKTN, FLRT1, FLVCR1, FOXA2, FOXE3, FOXG1, FOXRED1, FRMPD4, FSCN2, FXN, GABRA2, GABRA5, GABRB2, GABRD, GABRG2, GALC, GATA2, GATAD2B, GBA, GDF3, GDF6, GEMIN4, GFM2, GGCX, GJA1, GJC2, GLA, GLB1, GLI2, GLRX5, GMPPA, GMPPB, GNA11, GNAQ, GNAT1, GP1BA, GP1BB, GPAA1, GPR161, GRIA4, GRID2, GRIN2D, GRN, GTPBP2, GUCA1B, GUCY2D, HARS1, HCN1, HESX1, HGSNAT, HIKESHI, HIRA, HK1, HLA-A, HLA-B, HLA-DRB1, HNRNPK, HNRNPU, HSD17B10, HUWE1,
IBA57, IDH3A, IDH3B, IDS, IER3IP1, IFT140, IFT172, IFT88, IGBP1, IKBKG, IL10, IL11RA, IL12A, IL12A-AS1, IL23R, IMPDH1, IMPG2, INPP5E, INPP5K, INTS8, IQCB1,
ISCA2, ITM2B, IVNS1ABP, JMJD1C, KANSL1, KAT6A, KCNA2, KCNAB2, KCNB1, KCNC3, KCNJ13, KCTD7, KIAA0586, KIAA1549, KIF11, KIF14, KIF1A, KIF7, KIZ, KLC2, KLHL7, KLRC4, KMT2E, KNSTRN, KRAS, L2HGDH, LAMB1, LARGE1, LCA5, LDLR, LDLRAP1, LETM1, LHX3, LHX4, LIPT1, LRAT, LRP4, LRP5, LYRM7, MAFB, MAG, MAK, MAN2B1, MAP2K1, MAP2K2, MBTPS2, MC1R, MCOLN1, MECR, MED12, MED13, MEF2C, MEFV, MERTK, MFF, MFN2, MFRP, MFSD8, MGP, MICOS13, MICU1, MKS1, MMACHC, MMP14, MMP19, MMP2, MMUT, MOGS, MPDU1, MPDZ, MPLKIP, MRPS34, MSTO1, MT-ATP6, MT-CO3, MT-CYB, MT-ND2, MT-ND3, MT-ND4, MT-ND4L, MT-ND5, MT-ND6, MT-TL1, MT-TS1, MT-TS2, MTFMT, MTHFR, MTO1, MTPAP, MVK, MYO5A,
MYOC, MYT1L, NAA10, NAGA, NALCN, NARS2, NBAS, NDE1, NDP, NDUFA1, NDUFA10, NDUFA11, NDUFA12, NDUFA13, NDUFA2, NDUFA4, NDUFA6, NDUFA9, NDUFAF1, NDUFAF2, NDUFAF3, NDUFAF4, NDUFAF5, NDUFAF6, NDUFAF8, NDUFB10, NDUFB11, NDUFB3, NDUFB8, NDUFB9, NDUFS1, NDUFS2, NDUFS3, NDUFS4, NDUFS6, NDUFS7, NDUFS8, NDUFV1, NDUFV2, NECAP1, NEDD4L, NEK2, NELFA, NEU1, NF2, NFIX, NGLY1, NIPBL, NLRP3, NMNAT1, NOD2, NOTCH3, NR2E3, NR2F1, NR4A2, NRL, NSD2, NT5C2, NTNG1, NTRK2, NUBPL, NUP62, NUS1, OCA2, OFD1, OPA1, OPA3, OSTM1, OTX2, P4HTM, PANK2, PARS2, PAX2, PAX6, PCARE,

PCK1, PCLO, PCSK9, PCYT1A, PCYT2, PDE6A, PDE6B, PDE6G, PDGFB, PDHA1, PDHX, PDSS1, PET100, PEX1, PEX10, PEX11B, PEX12, PEX13, PEX14, PEX16, PEX19, PEX2, PEX26, PEX3, PEX5, PEX6, PIEZO2, PIGB, PIGU, PIK3CA, PIK3CD, PISD, PLA2G6, PLAA, PLEKHM1, PLK4, PLP1, PMPCB, PNPLA6, POGZ, POLG, POLR3A, POLR3B, POMGNT1, POMGNT2, POMK, POMT1, POMT2, PORCN, POU1F1, POU3F4, PPP1CB, PPP2R1A, PPP3CA, PPT1, PRCD, PRDM16, PRICKLE3, PRKAR1A, PROKR2, PROM1, PROP1, PRPF3, PRPF31, PRPF4, PRPF6, PRPF8, PRPH2, PRPS1, PRUNE1, PSAP, PSMD12, PTCD3, PTEN, PTF1A, PTGS1, PTGS2, PTPN22, PTPN23, PUF60, RAB11B, RAB18, RAB23, RAB3GAP1, RAB3GAP2, RBM10, RBP3, RD3, RDH12, RECQL4, REEP6, RERE, RFC1, RGR, RHO, RHOA, RIMS2, RLBP1, RLIM, RNF113A, RNF135, RNF216, ROBO1, ROM1, RP1, RP1L1, RP2, RP9, RPE65, RPGR, RPGRIP1, RPGRIP1L, RPIA, RPS6KA3, RREB1, RSPO2, RTN4IP1, RTTN, RXYLT1, SAG, SALL2, SALL4, SARDH, SCAPER, SCN3A, SCN8A, SCO2, SCYL1, SCYL2, SDHA, SEC23A, SEC24C, SEC31A, SELENOI, SEMA3E, SEMA4A, SERAC1, SH2B1, SH3BP2, SIL1, SIX6, SKI, SLC13A5, SLC19A2, SLC19A3, SLC1A2, SLC25A19, SLC25A46, SLC29A3, SLC38A8, SLC39A14, SLC44A1, SLC45A2, SLC52A2, SLC6A9, SLC7A14, SMARCB1, SMARCE1, SMO, SMOC1, SNAP29, SNRNP200, SNX10, SON, SOST, SOX2, SOX3, SOX5, SPATA7, SPG7, SPINT2, SPOP, SRD5A3, SSBP1, ST3GAL5, STAG2, STAMBP, STAT4, STT3B, STXBP1, SUFU, SUMF1, SURF1, SVBP, SYNGAP1, SYNJ1, SZT2, TACO1, TANC2, TANGO2, TASP1, TBC1D20, TBC1D24, TBC1D7, TBCD, TBCE, TBX1, TCF20, TCIRG1, TCTN2, TERT, TFG, TGFB1, THG1L, TIMM50, TIMM8A, TIMMDC1, TK2, TLR4, TMEM107, TMEM126A, TMEM126B, TMEM216, TMEM231, TMEM237, TMEM63A, TMEM67, TMEM98, TMTC3, TNFRSF11A, TNFRSF11B, TNFSF11, TOE1, TOPORS, TOR1A, TP53, TPI1, TRAF7, TRAK1, TRAPPC11, TRAPPC12, TRAPPC4, TRIT1, TRMT1, TRNP1, TRNT1, TSC1, TSC2, TSFM, TTC8, TUB, TUBA8, TUBB3, TUBB4A, TUBGCP2, TUBGCP4, TUBGCP6, TULP1, TWIST1, TWNK, TXN2, TYR, TYRP1, UBA5, UBAC2, UBE3B, UCHL1, UFD1, UQCRFS1, USH2A, USP45, VCAN, VHL, VPS11, VPS13B, VPS33A, VPS53, WARS2, WASHC5, WDFY3, WDPCP, WDR11, WDR4, WDR45, WDR73, WFS1, WNT3, WT1, WWOX, XPA, XPC, XYLT1, XYLT2, YAP1, YME1L1, YWHAG, ZEB2, ZIC1, ZMIZ1, ZNF408, ZNF513, ZNF592, ZNHIT3
