# Supplementary material for: Case Report: A Novel Mutation in the Mitochondrial MT-ND5 Gene Is Associated With Leber Hereditary Optic Neuropathy (LHON)
Source: Front Neurol. 2021 Mar 25;12:652590. doi: 10.3389/fneur.2021.652590 (PMC8027302; doi:10.3389/fneur.2021.652590)
Supplement: Supplementary file 2 [file Data_Sheet_2.pdf]

CARE case report guidelines

CARE Checklist of information to include when writing a case report

(cc) BY-NC-ND

✓

Topic	Item	Checklist item description	Reported on Line
**Title**	1	The diagnosis or intervention of primary focus followed by the words "case report"	1-2
**Key Words**	2	2 to 5 key words that identify diagnoses or interventions in this case report, including "case report"	25-26
**Abstract (no references)**	3a	Introduction: What is unique about this case and what does it add to the scientific literature?	40-78
	3b	Main symptoms and/or important clinical findings	81-175
	3c	The main diagnoses, therapeutic interventions, and outcomes	168-175
	3d	Conclusion—What is the main "take-away" lesson(s) from this case?	211-213
**Introduction**	4	One or two paragraphs summarizing why this case is unique (**may include references**)	211-213
**Patient Information**	5a	De-identified patient specific information.	81-175
	5b	Primary concerns and symptoms of the patient.	81-175
	5c	Medical, family, and psycho-social history including relevant genetic information	81-175
	5d	Relevant past interventions with outcomes	not applicable
**Clinical Findings**	6	Describe significant physical examination (PE) and important clinical findings.	81-175
**Timeline**	7	Historical and current information from this episode of care organized as a timeline	81-175
**Diagnostic Assessment**	8a	Diagnostic testing (such as PE, laboratory testing, imaging, surveys).	81-175
	8b	Diagnostic challenges (such as access to testing, financial, or cultural)	not applicable
	8c	Diagnosis (including other diagnoses considered)	168
	8d	Prognosis (such as staging in oncology) where applicable	not applicable
**Therapeutic Intervention**	9a	Types of therapeutic intervention (such as pharmacologic, surgical, preventive, self-care)	168-170
	9b	Administration of therapeutic intervention (such as dosage, strength, duration)	169
	9c	Changes in therapeutic intervention (with rationale)	not applicable
**Follow-up and Outcomes**	10a	Clinician and patient-assessed outcomes (if available)	170-175
	10b	Important follow-up diagnostic and other test results	84-175
	10c	Intervention adherence and tolerability (How was this assessed?)	not assessed/not applicable
	10d	Adverse and unanticipated events	not applicable
**Discussion**	11a	A scientific discussion of the strengths AND limitations associated with this case report	190-210
	11b	Discussion of the relevant medical literature **with references**.	41-78, 177-210
	11c	The scientific rationale for any conclusions (including assessment of possible causes)	177-210
	11d	The primary "take-away" lessons of this case report (without references) in a one paragraph conclusion	211-213
**Patient Perspective**	12	The patient should share their perspective in one to two paragraphs on the treatment(s) they received	not applicable
**Informed Consent**	13	Did the patient give informed consent? Please provide if requested	Yes ☒ No ☐
